# Adjunct low-dose ketamine infusion vs standard of care in mechanically ventilated critically ill patients at a Tertiary Saudi Hospital (ATTAINMENT Trial): study protocol for a randomized, prospective, pilot, feasibility trial

**DOI:** 10.1186/s13063-020-4216-4

**Published:** 2020-03-20

**Authors:** Mohammed Bawazeer, Marwa Amer, Khalid Maghrabi, Kamel Alshaikh, Rashid Amin, Muhammad Rizwan, Mohammad Shaban, Edward De Vol, Mohammed Hijazi

**Affiliations:** 1 Department of Critical Care Medicine (MBC 94), King Faisal Specialist Hospital and Research Center, P.O Box 3354, Riyadh, 11211 Saudi Arabia; 2 Pharmaceutical Care Division (MBC 11), King Faisal Specialist Hospital and Research Center, P.O Box 3354, Riyadh, 11211 Saudi Arabia; 3 Department of Biostatistics, Epidemiology and Scientific Computing, King Faisal Specialist Hospital and Research Center, P.O Box 3354, Riyadh, 11211 Saudi Arabia

**Keywords:** Ketamine, Standard of care, Sedation, Critically ill, Mechanical ventilation, ATTAINMENT, Propofol, Fentanyl, Midazolam, Delirium, Vasopressors

## Abstract

**Background:**

A noticeable interest in ketamine infusion for sedation management has developed among critical care physicians for critically ill patients. The 2018 Pain, Agitation/sedation, Delirium, Immobility, and Sleep disruption guideline suggested low-dose ketamine infusion as an adjunct to opioid therapy to reduce opioid requirements in post-surgical patients in the intensive care unit (ICU). This was, however, rated as conditional due to the very low quality of evidence. Ketamine has favorable characteristics, making it an especially viable alternative for patients with respiratory and hemodynamic instability. The Analgo-sedative adjuncT keTAmine Infusion iN Mechanically vENTilated ICU patients (ATTAINMENT) trial aims to assess the effect and safety of adjunct low-dose continuous infusion of ketamine as an analgo-sedative compared to standard of care in critically ill patients on mechanical ventilation (MV) for ≥ 24 h.

**Methods/design:**

This trial is a prospective, randomized, active controlled, open-label, pilot, feasibility study of adult ICU patients (> 14 years old) on MV. The study will take place in the adult ICUs in the King Faisal Specialist Hospital and Research Center (KFSH&RC), Riyadh, Saudi Arabia, and will enroll 80 patients. Patients will be randomized post-intubation into two groups: the intervention group will receive an adjunct low-dose continuous infusion of ketamine plus standard of care. Ketamine will be administered over a period of 48 h at a fixed infusion rate of 2 μg/kg/min (0.12 mg/kg/h) in the first 24 h followed by 1 μg/kg/min (0.06 mg/kg/h) in the second 24 h. The control group will receive standard of care in the ICU (propofol and/or fentanyl and/or midazolam) according to the KFSH&RC sedation and analgesia protocol as clinically appropriate. The primary outcome is MV duration until ICU discharge, death, extubation, or 28 days post-randomization, whichever comes first.

**Discussion:**

The first patient was enrolled on 1 September 2019. As of 10 October 2019, a total of 16 patients had been enrolled. We expect to complete the recruitment by 31 December 2020. The findings of this pilot trial will likely justify further investigation for the role of adjunct low-dose ketamine infusion as an analgo-sedative agent in a larger, multicenter, randomized controlled trial.

**Trial registration:**

ClinicalTrials.gov: NCT04075006. Registered on 30 August 2019. Current controlled trials: ISRCTN14730035. Registered on 3 February 2020.

## Background

Sedation and analgesia management are both integral components of care in the intensive care unit (ICU). Although benzodiazepines have been the mainstay therapy for sedation in critically ill patients, their use has declined in recent years, with favoring of non-benzodiazepines, such as propofol and dexmedetomidine. This change in practice is based on studies demonstrating the association between the sustained use of benzodiazepines and increased mechanical ventilation (MV) duration, ICU length of stay (LOS), and development of delirium. A paradigm shift has therefore occurred in the management of patients’ sedation in the ICU. Maintenance of light levels of sedation in adult patients in the ICU has been recommended to improve patient clinical outcomes, such as shorter duration of MV and shorter ICU LOS [1].

A noticeable interest in ketamine infusion for sedation management in critically ill patients has developed among critical care physicians [2]. The 2018 Pain, Agitation/sedation, Delirium, Immobility, and Sleep disruption (PADIS) guideline suggested low-dose ketamine as an adjunct to opioid therapy for reducing opioid consumption in post-surgical adults admitted to the ICU (i.e., conditional recommendation, very low quality of evidence) [1]. In a single-center, double-blind, randomized controlled trial (RCT) of 93 ICU post-abdominal surgery patients, adjunctive ketamine was associated with a reduced intake of morphine. However, there were no differences in patients’ self-reported pain intensity [3]. Ketamine was administered as 0.5 mg/kg intravenous (IV) push followed by infusion of 2 μg/kg/min (0.12 mg/kg/h) × 24 h, then 1 μg/kg/min × 24 h (0.06 mg/kg/h). The incidence of side effects (i.e., nausea, delirium, hallucination, hypoventilation, pruritus, and sedation) did not differ between the ketamine and opioid-alone groups. Based on this generally positive ICU RCT, the 2018 PADIS panel made a conditional recommendation for the use of low-dose ketamine as an adjunct to opioids to optimize acute post-operative pain management in critically ill adults (refer to Supplementary Table 1: Previous ketamine trials in the ICU setting) [2–11].

Because of an increased focus on ensuring that pain is appropriately controlled in patients before using sedative-hypnotic medications (also known as the analgo-sedation approach), ketamine has gained attention for its unique pharmacologic properties that could address both the analgesic and sedative requirements. Ketamine could result in decreased duration of MV while providing optimal levels of sedation [12]. Similar to dexmedetomidine, ketamine has a non-GABAergic mechanism of action [13]. It induces rapid sedation and analgesia through dual mechanisms mediated by inhibition of the *N*-methyl-d-aspartate receptor and activation of the opioid μ- and κ-receptors [14]. Ketamine is also Saudi Food and Drug Authority (FDA)-approved for the induction of anesthesia and has been used for acute and chronic pain in sub-anesthetic dose, post-operative opioid sparing, rapid sequence intubation, and procedural sedation and analgesia [15]. Additionally, ketamine has favorable characteristics, including bronchodilation, preservation of cardiac output, increase in blood pressure, minimal effects on bowel motility, and maintaining of respiratory drive and airway reflexes while actively weaning from MV; these features make it an especially viable alternative for patients with respiratory and hemodynamic instability [12].

Although commonly used sedatives are effective, they have side effects including benzodiazepine-associated delirium, opioid-induced constipation, and the negative hemodynamic effect caused by propofol and dexmedetomidine [1, 16–18]. The most frequently observed adverse effects associated with ketamine when used to maintain sedation include tachycardia (6.7%), hypertension (6%), paradoxical agitation (up to 20%), and hypersalivation (12%) [12]. Although there is limited literature on adults, as many as 57% of *pediatric* patients who receive ketamine for continuous sedation experience the emergence phenomenon, including vivid hallucinations and delirium during or after ketamine use [19]. When ketamine is used for procedural sedation in adults (usually administered as a relatively high dose, 1–2 mg/kg repeated q5–15 min to maximum 100 mg), up to 20% of patients may develop the emergence phenomenon [20]. Risk factors for delirium with ketamine include prior history of psychiatric disorders, dementia, and the use of a high dose in procedural sedation [20]. The development of the emergence phenomenon can cause patients to transiently require higher amounts of other sedatives, usually benzodiazepines. However, ketamine-based analgo-sedation in MV patients administered as a sub-anesthetic/sub-dissociative/low dose results in similar numbers of delirium- and coma-free days as those in non-ketamine-based regimens, as shown in a retrospective cohort study conducted by Shurtleff et al. at an academic medical center [8]. Ketamine infusion in this trial was 5 *μ* g/kg/min (0.3 mg/kg/h) titrated using 5 *μ* g/kg/min every 5 min up to a maximum of 25 *μ* g/kg/min (1.5 mg/kg/h). The authors found that the number of days alive without delirium or coma was 6 days (interquartile range [IQR] 2–9 days) with ketamine and 4 days (IQR 3–7 days) with a non-ketamine medication (*P* = 0.351). Delirium occurred in 29 of the 39 patients (74%) with ketamine and in 34 of the 40 patients (85%) with the non-ketamine drug (*P* = 0.274). Similarly, the RCT cited by the 2018 PADIS guideline showed that the incidence of side effects (i.e., delirium and hallucinations) did not differ between the ketamine and opioid-alone groups [3].

At King Faisal Specialist Hospital and Research Center (KFSH&RC), continuous infusions of sedatives and analgesics are prescribed at the physician’s discretion and titrated to achieve Richmond Agitation-Sedation Scale (RASS) and pain scores; the infusions are performed with a nurse-driven protocol. The protocol promotes analgesia-first sedation (with fentanyl) and recommends propofol as the first-line agent when sedation is required. Patients receive a daily spontaneous awakening trial (SAT) paired with a spontaneous breathing trial (SBT). The RASS and the Confusion Assessment Method for the ICU (CAM-ICU) are routinely used to assess the level of sedation and the presence of delirium, respectively. Ketamine, registered by the Ministry of Health of Saudi Arabia, is a KFSH&RC hospital formulary medication and is listed in the KFSH&RC ICU pain and sedation protocol as an option for patients with severe bronchospasm. However, the order or the combination that could be most effective with ketamine is unclear (refer to Supplementary Figure 1: KFSH&RC new sedation protocol for adult ICUs).

As stated previously, the 2018 PADIS guideline listed ketamine as a conditional recommendation, with very low quality of evidence (limited high-level evidence). Most trials listed in Supplementary Table 1 are in surgical ICU settings, retrospective in nature, or are RCTs focused on comparing ketamine to placebo or two study drugs (e.g., ketamine vs opioid). However, most patients in the ICU are sedated with a combination of drugs. Moreover, most trials had a limited focus on patient-centered outcomes, such as duration of MV or ICU LOS, as the primary outcome favoring surrogate outcomes, such as sedation scores and changes in analgesics and sedatives [2–11]. To help further delineate ketamine’s role as a maintenance analgo-sedation agent in the ICU, further RCTs need to be conducted to compare the effects of ketamine to those of other analgesics and sedatives on reducing the duration of MV, ICU LOS, and delirium occurrence. Recently, there was a prospective, double-blinded, multicenter RCT (KeMiMof) in critically ill patients > 12 years old and requiring sedation for > 24 h in the ICU, in Uganda. Patients were randomized to receive either ketamine-midazolam or morphine-midazolam given as premixed 50-ml syringes for infusion. The primary outcome measures were duration of MV, incidence of hypotension, and incidence of delirium. The trial was terminated on 28 August 2019 with pending results. Limitations of this trial are the use of premixed syringes, which are not typically used in adult ICU sedation practice, and the focus on comparing two study drugs (ketamine vs morphine) [21].

Robust clinical outcome data and comprehensive assessments of adverse events (AEs) associated with ketamine use in mechanically ventilated patients are limited, leaving a significant knowledge gap, which has been reflected in the wide variation in the use of ketamine as a sedative agent in ICUs. This is also highlighted in a recent systematic review and meta-analysis by Manasco et al. [22]. Therefore, we propose a prospective, randomized, active controlled, open-label, pilot, feasibility study to assess the effect and safety of *A*nalgo-sedative adjunc*T* ke*TA*mine *I*nfusion i*N M*echanically v*ENT*ilated ICU patients (the ATTAINMENT trial) compared to standard of care alone. We hypothesized that low-dose ketamine infusion will reduce the duration of MV with an acceptable safety profile compared to standard of care. The findings of this pilot trial will likely justify further investigations on the role of adjunct low-dose ketamine infusion as an analgo-sedative and inform the design of a large multicenter RCT with sufficient power to detect differences in clinical outcomes.

### Study objectives

#### The primary objective

The primary objective is to study the feasibility and effect of adjunct low-dose ketamine infusion on MV duration compared to the standard of care alone in critically ill patients.

#### The secondary objectives

Secondary objectives are to study the effect of adjunct low-dose ketamine infusion on the following:

1. The cumulative dose of pain and sedative medications
2. The incidence of dexmedetomidine use post-randomization
3. The number of patients within RASS and pain score goals
4. The hemodynamic status in terms of vasopressor therapy requirement
5. ICU and hospital LOS
6. Tracheostomy, unplanned extubation, and re-intubation rates
7. The incidence of delirium and rate of positive CAM-ICU assessment
8. The rate of antipsychotic use for ICU-acquired delirium
9. The rate of hypersalivation and frequent suctioning
10. The rate of using physical restraint
11. Mortality rate at 28 days.

## Methods

### Design overview

The ATTAINMENT trial is a prospective, randomized, open-label, active controlled, parallel group, pilot, feasibility, phase 3 study of adult patients admitted to the KFSH&RC adult ICUs, Riyadh, Saudi Arabia. This trial is approved by the Institutional Review Board (IRB) of the KFSH&RC. The trial is registered in ClinicalTrials.gov: NCT04075006, current controlled trials: ISRCTN14730035, and Saudi Food and Drug Authority: SCTR #19063002. The protocol adheres to the Standard Protocol Items: Recommendations for Interventional Trials (SPIRIT) guidelines (see Supplementary File 1 and Supplementary Table 2).

### Eligibility and enrollment

The inclusion criteria are as follows:

- Adult patients (> 14 years old) on MV admitted to one of the following ICUs: medical, surgical, or transplant/oncology ICU
- Intubated within the previous 24 h
- Expected to require MV for more than 24 h
- Expected to be on the KFSH&RC ICU sedation and pain protocol
- No objection from the ICU attending physician for enrollment
  The exclusion criteria are:
- Patients with a history of dementia or psychiatric disorders or those on any antipsychotic or antidepressant medications at home
- Pregnancy
- Age < 14 years old
- Expected to need MV < 24 h
- Known hypersensitivity to ketamine
- Patients with expected targeted RASS score of − 5, e.g., patients on continuous infusion neuromuscular blockade
- Patients on dexmedetomidine as the primary sedative prior to randomization
- Patients with cardiogenic shock, acute decompensated heart failure, or myocardial infarction
- History of end-stage liver failure (Child-Pugh score C)
- Proven or suspected primary neurological injury (traumatic brain injury, ischemic stroke, intracranial hemorrhage, spinal cord injury, anoxic brain injury, brain edema)
- Patients with persistent heart rate (HR) > 150 beats per minute (bpm) or systolic blood pressure (SBP) > 180 mmHg
- Patients identified as Do Not Resuscitate (DNR) or those expected to die within 24 h
- Patients on extracorporeal membrane oxygenation (ECMO)
- Patients with refractory status epilepticus who are receiving ketamine infusion
- Proven or suspected status asthmaticus.

### Informed consent

The study is conducted according to Good Clinical Practice guidelines. The study protocol as well as the informed consent have been approved by the Research Ethics Committee (REC) and Clinical Research Committee (CRC) at KFSH&RC with Research Advisory Council (RAC) number 2191 187. Once an eligible patient is identified, study investigators start the consenting process to explain the objectives of the trial and its potential risks and benefits to the patient’s surrogate decision-maker. A verbal consent from a guardian/next of kin over the phone is considered to allow randomization and initiation of timely intervention when written authorization cannot be secured in sufficient time (within 24 h of intubation). Verbal consent is documented in medical records to indicate the research subject’s acceptance to participate in the study. A prospective written consent is obtained thereafter from the patient (if extubated) or the patient’s guardian/next of kin once they become available.

### Trial interventions

**Fig. 1:**
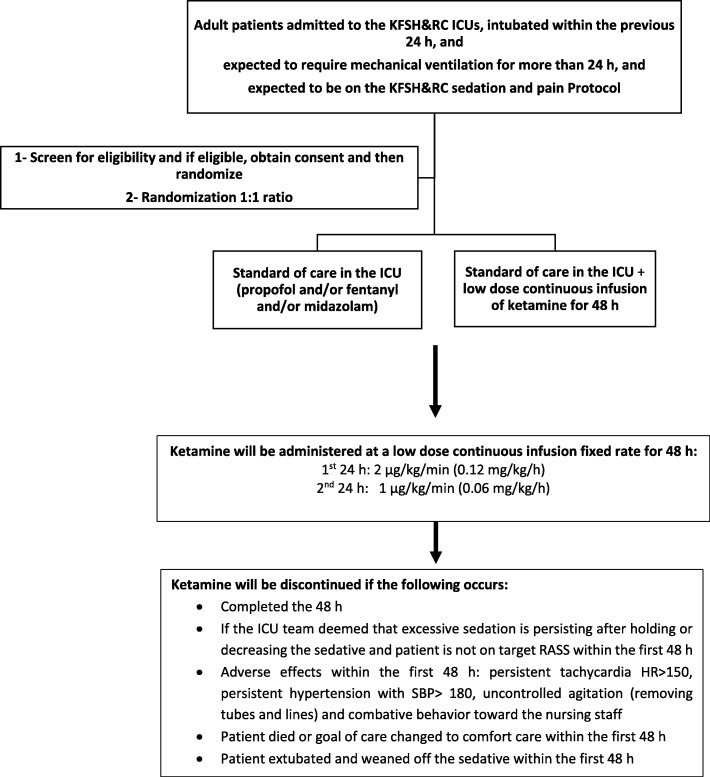
Study methodology

Patients will be randomized into two groups: the intervention group will receive an adjunct low-dose continuous infusion of ketamine plus the standard of care in the ICU. Ketamine will be administered over a 48-h period at a fixed infusion rate of 2 μg/kg/min (0.12 mg/kg/h) for the first 24 h followed by 1 μg/kg/min (0.06 mg/kg/h) in the second 24 h. The control group will receive the standard of care in the ICU, where propofol and/or fentanyl and/or midazolam will be given according to the KFSH&RC ICU sedation and analgesia protocol as clinically appropriate. Other aspects of care in both groups, including RASS goal, SAT, SBT, mobilization, and non-pharmacological interventions to promote comfort and facilitate sleep, will be left to the discretion of the ICU attending physician. Please refer to Fig. 1: study methodology.

### Stopping guidelines for the intervention

The intervention may be stopped in the following situations (see Table 1 for more details):

- The ICU team deemed that excessive sedation is persisting after holding or decreasing the other sedatives (propofol and/or fentanyl and/or midazolam) and the patient is not in target RASS.
- Adverse effects: persistent tachycardia with HR > 150 for ≥ 3 h, persistent hypertension with SBP > 180 for ≥ 3 h, uncontrolled agitation (removing tubes and lines) and combative behavior.
- Patient died or goals of care changed to comfort care.
- Patient is weaned off sedation and/or extubated.

**Table 1:** Intervention stopping rules and protocol deviation

Events	Action regarding the intervention (ketamine)	Action regarding the study procedure (data collection and data analysis)
Completed 48 h	Ketamine will be discontinued (intended duration for this trial is 48 h). Continuation of ketamine or other analgesics and sedatives for more than 48 h will be left to the treating physicians, but will not be related to the research purpose	Subject will be included in the data analysis
Positive CAM-ICU score for delirium and hallucination within the first 48 h	Ketamine will be continued, and delirium treatment (non-pharmacological and antipsychotic use) will be left to the treating physicians. In cases of uncontrolled agitation (removal of tubes and lines and combative behavior) within the first 48 h, ketamine will be discontinued (refer to protocol deviation below)	Subject will be included in the data analysis (safety outcome data)
Use of physical restraint within the first 48 h	Ketamine will be continued unless uncontrolled agitation (removal of tubes and lines and combative behavior) within the first 48 h, in which case ketamine will be discontinued (refer to protocol deviation below)	Subject will be included in the data analysis (safety outcome data)
Hypersalivation and frequent suctioning within the first 48 h	Ketamine will be continued and management of hypersalivation will be left to the treating physicians	Subject will be included in the data analysis
**Protocol deviation (patient did not complete the intended duration of the trial (i.e., 48 h)**		
Patient or proxies withdraw consent	Ketamine will be discontinued	All information will be removed and not included in the analysis (modified intention-to-treat principle)
Patient extubated and sedation weaned off within the first 48 h	Ketamine will be discontinued	Subject will be included in the data analysis
If the ICU team believed the patient is not in target for RASS within the first 48 h	**When the patient is deemed to be excessively sedated** after receiving ketamine and other sedatives (propofol and/or fentanyl and/or midazolam), the other sedatives will be held first (or decreased) and ketamine will be continued until the subject reaches the team’s desired RASS goal. In situations where excessive sedation persisted and the patient is not yet in target RASS, then ketamine will be discontinued **When the patient is deemed to be agitated** after receiving ketamine and other sedatives (propofol and/or fentanyl and/or midazolam), the other sedatives will be increased, use as needed boluses, or add dexmedetomidine. The decision to continue or discontinue ketamine infusion will be left to the discretion of the treating physicians	Subject will be included in the data analysis
Persistent tachycardia with HR > 150 for > 3 h within the first 48 h	If the ICU treating physicians believes that ketamine is the primary causative factor, ketamine will be discontinued and patient will be followed up for 24 h. Detailed documentation will be carried out in the medical record for adverse event, severity of event, recovery from event, group allocation, and relation to study protocol	Subject will be included in the data analysis
Hypertension with SBP > 180 for > 3 h within the first 48 h		Subject will be included in the data analysis
Uncontrolled agitation (pulling off tubes and lines) within the first 48 h		Subject will be included in the data analysis
Combative behavior within the first 48 h		Subject will be included in the data analysis
Patient died^a^ or goal of care changed to comfort care within the first 48 h	Ketamine will be discontinued	Subject will be included in the data analysis (safety outcome data)
Physician decline after randomization	Ketamine will be discontinued	Subject will be included in the data analysis

^a^In cases of death (either within the first 48 h, until ICU or hospital discharge, or 28 days after randomization, whichever comes first), detailed documentation will be carried out in the medical record for the cause of death, group allocation, and relation to study protocol

### Randomization

Patients will be randomly assigned to one of two study groups in a 1:1 ratio by a computer-generated randomization list created by an independent biostatistician; no stratification will be performed. Our initial screening and eligibility assessment is done by bedside ICU nurses who are blinded to treatment assignment. To further ensure allocation concealment, access to the randomization will be restricted to a pharmacist (third party and not part of the study) to whom principal investigators refer at a distance (by telephone) to know the assigned treatment. The study investigators and study participants during the recruitment and consenting process will be blinded to the treatment assignment. Once the consenting process is complete, the principal investigators will contact the pharmacist (third party) for patient allocation and initiation of the trial intervention. Group allocation will be concealed until after randomization.

### Duration of the intervention

**Fig. 2:**
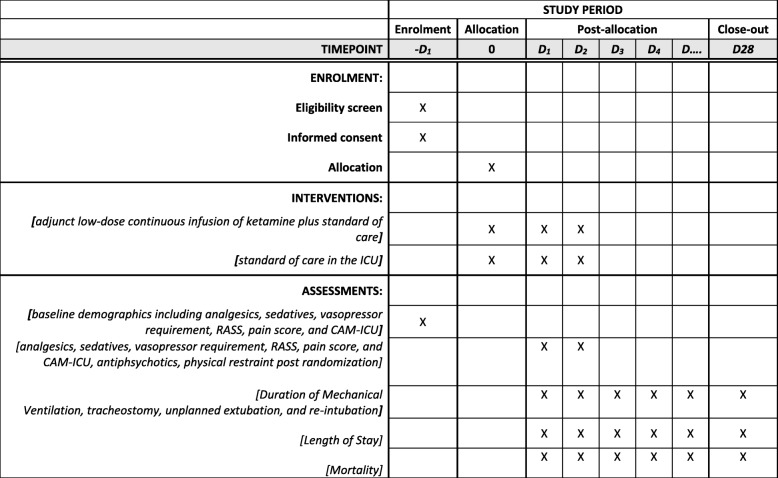
Schedule of enrollment, interventions, and assessments for Analgo-sedative adjuncT keTAmine Infusion iN Mechanically vENTilated ICU patients (ATTAINMENT trial)

The study interventions will continue for 48 h from the time of randomization. Patients and medical charts will be followed at baseline prior to randomization and at 24 h and 48 h post-randomization. Medical charts will also be followed to document the outcomes at 28 days, or until death, whichever comes first. Please refer to Fig. 2 for the schedule of enrollment, interventions, and assessments.

### Minimizing bias

#### Blinding

The study investigators and study participants during the recruitment and consenting process will be blinded to the treatment assignment. Once the trial intervention starts, the treating team and study investigators will not be blinded to the trial intervention for practical and safety purposes (open label). The study statistician is blinded to the treatment allocation, and the study investigators will remain blinded to the results until the conclusion of the study.

#### Minimizing contamination

The principal investigators will ensure enrollment of patients as quickly as possible after 24 h post-intubation. Patients intubated for more than 24 h will be excluded to eliminate early contamination or confounders.

### Outcomes and follow-up

#### Primary outcome

The primary outcome is median duration of MV: the number of calendar days from intubation date to extubation date, until ICU discharge, death, or 28 days post-randomization, whichever comes first. This outcome was chosen as a patient-centered outcome and based on the mechanistic plausibility data that showed ketamine possibly has a bronchodilatory effect and maintains respiratory drive and airway reflexes [9, 12, 13]. Because duration of MV is highly influenced by mortality, the median ventilator-free days to day 28 post-randomization will be calculated as a co-primary outcome [23]. See the statistical methods in “Data analysis.”

#### Secondary clinical outcomes

1. Proportion and median cumulative dose of pain and sedative medications in the first 48 h after randomization
2. Proportion of patients started on dexmedetomidine 48 h after randomization
3. Proportion of patients achieving the RASS goal and pain score goal within the first 48 h after randomization
4. Proportion and median vasopressor requirements in the first 48 h after randomization
5. Median change in mean arterial pressure (MAP) and HR in the first 48 h after randomization
6. ICU and hospital LOS: number of calendar days (median, IQR) from randomization to discharge date from the ICU or hospital
7. Proportion of tracheostomy, unplanned extubation (self-extubation), and re-intubation within 28 days post-randomization
8. Proportion of patients starting on antipsychotics and positive CAM-ICU score to assess the incidence of delirium 48 h after randomization. The presence of delirium will also be confirmed through a psychiatrist consultation
9. Proportion of physical restraint 48 h after randomization
10. Proportion of patients with frequent suctioning in the first 48 h after randomization (defined as interval between suctioning episodes 2 h or less)
11. Mortality rate at the time of hospital discharge or 28 days after randomization, whichever comes first.

#### Secondary feasibility outcomes:

1. Proportion of screened patients
2. Proportion of eligible patients enrolled
3. Enrollment rate (i.e., number of enrollments per month)
4. Protocol compliance.

#### Data management

Data will be collected in the KFSH&RC Research Electronic Data Capture (REDCap) platform. Each subject will be given a unique subject ID number (database numbers and all identifiers will be removed). A subject ID key will be used to match the subjects’ Medical Record Numbers and will be kept in a password-protected file that is accessible to the principal investigators. Access to the RedCap data will be limited to the principal investigators and co-investigators involved in data collection only. Access to REDCap requires authentication (username and password) for secure maintenance of the data. All investigators are KFSH&RC employees and have access to the electronic medical record (Power Chart). All collected information will be stored in a secure manner, and all patient data will be kept confidential. To ensure consistency in data collection, training sessions will be held by the principal investigators for all research co-investigators involved in data collection prior to study commencement. Additionally, the principal investigators will conduct educational sessions for ICU physicians and ICU nurses, which will include the study protocol, and periodic follow-up educational sessions to provide feedback and ensure optimal compliance with the study protocol. There will be periodic internal audits of data entry accuracy and compliance by the principal investigators. This will allow us to identify any protocol deviations and provide an opportunity for feedback to the co-investigators involved in data entry. Range edits and value checks will be incorporated into the REDCap software to minimize the potential for data entry errors. Moreover, printed copies of de-identified Case Report Forms will be submitted to the RAC at the KFSH&RC any time upon committee request and will be reviewed upon the receipt of the progress report by 4 June 2020 (the date specified originally upon IRB approval of the study protocol).

The following data will be collected: age, gender, weight, mode of MV at baseline, percentage of renal replacement therapy at baseline, lactate level at baseline, and severity of illness as estimated by Sequential Organ Failure Assessment (SOFA) score and Acute Physiology and Chronic Health Evaluation (APACHE II) score, with higher scores indicating higher severity of illness [24]. Moreover, we will collect ICU type, baseline analgesics, sedatives, vasopressor requirements, and PRE-DELIRIC Delirium Risk Score, which is a delirium prediction model specifically designed for adult critical care patients 24 h after ICU admission. This model will be used to predict the factors that may influence delirium risk prior to randomization [25]. We will also collect RASS, pain, and CAM-ICU scores at baseline and at 24 and 48 h post-randomization. The RASS is a scale used to assess the depth of sedation on a scale of − 5 to + 4, with a negative value indicating deeper sedation and positive values indicating increased agitation [1]. The CAM-ICU is a valid and reliable delirium assessment tool. Patients with a RASS score of − 3 or lower will be excluded from CAM-ICU assessment, as they cannot participate in the exam [26]. We will calculate the modified Clinical Pulmonary Infection Score (CPIS) to differentiate secretions caused by patients’ underlying lung pathology (ventilator-associated pneumonia [VAP]) vs ketamine-associated hypersalivation [27]. We will also record the proportion of eligible participants enrolled, rates of recruitment, protocol deviations, and AEs.

### Data analysis

The sample size calculation associated with the specified (required) number of patients to be recruited is based on the study by Buchheit et al. [9]. In their study, the median time from initiation of ketamine to extubation was 1.44 days (IQR 0.58–2.66). As time from initiation of ketamine to extubation is bounded below by zero, and assuming that the distribution is skewed toward larger times, a lognormal distribution was assumed to be appropriate for modeling such times.

We additionally assumed that the distribution of times from intubation to extubation for patients who are not treated with ketamine is lognormal, with median 2.44 and IQR from 1.60 to 4.00.

The hypothesis of interest is H0: m1 = m2 vs Ha: m1 ≠ m2, where m1 is the median time for those treated with ketamine, and m2 is the median time for those not treated with ketamine. Here the times are distributed with lognormal distributions, and the respective IQRs are 0.58–2.66 for ketamine-treated patients and 1.60–4.00 for those not treated with ketamine. A simulation analysis was carried out with 35 simulated patient times under each of the above two distributional scenarios, i.e., 35 intubation to extubation times under a lognormal of 1.44 and 0.58–2.66 (median and IQR) and 35 intubation to extubation times under a lognormal of 2.44 and 1.60–4.00 (median and IQR). This was followed by calculation of the level of significance by the Wilcoxon rank sum test (i.e., the associated *P* value). By repeating this simulation 10,000 times, 80.24% of the simulations had a *P* value less than 0.05. This shows that the power of a design with 35 ketamine-treated and 35 non-ketamine-treated patients should (with at least 80% probability) demonstrate that the time from intubation to extubation is one day less for those treated with ketamine. It is recognized that non-compliance and dropouts may occur. Hence, the study has been designed to recruit 40 ketamine-treated and 40 untreated subjects for analyses (i.e., total sample size 80). The median ventilator-free days will be calculated as calendar days with no ventilator support to day 28 post-randomization. Participants who die before day 28 are assigned zero free days. Data will be analyzed using the modified intention-to-treat principle and will comprise data from all patients who undergo randomization, with the exception of those who withdraw consent, have an unknown primary outcome, or are identified as ineligible after randomization. The Shapiro-Wilk test for normality will be used to assess the distribution of all outcome variables. Chi-square and *t* tests (or Wilcoxon rank sum) will be used to compare categorical data and continuous data, respectively. All data will be presented as median and IQR, if not normally distributed (or count and percentages, if categorical). Univariate and multivariate regression analyses will be used to identify risk factors and predictors for delirium. Statistically significant factors in the univariate analysis (≤ 0.05) will be included in the multivariate analysis. Adjustments for the analysis will be accounted for with the Bonferroni technique. A prespecified sub-group analysis of the primary outcome will be conducted on the following variables:

- Age > 60 vs age < 60
- SOFA score > 10 vs < 10
- APACHE II > 20 vs < 20
- Ratio of the partial pressure of arterial oxygen to the fraction of inspired oxygen (PF ratio > 150 vs PF ratio < 150)
- Surgical vs medical admission.

We will strive to obtain full data on every patient to allow an intention-to-treat analysis. If there is missing information as patients withdraw from the study before completion of the follow-up period, it will be handled in the normal fashion of survival analysis (censored observation). Imputation (based on regression model) will be considered in case of incomplete information about key covariates. Sensitivity analyses with such excluded patients will be conducted and compared with an imputed values model. Each of the analyses will be redone to test each hypothesis and verify the robustness of the conclusion. Statistical analyses will be performed using SAS/JMP, v.14.1 (SAS Institute, Cary, NC, USA). The study statistician is blinded to the treatment allocation, and study investigators will remain blinded to the results until the conclusion of the study.

### Trial administration and Safety Monitoring Committee

The principal investigators will meet weekly to perform periodic internal audits of data accuracy, review enrollment rates, and oversee and coordinate the study in general. This will allow them to identify any protocol deviations and provide an opportunity for feedback to the other co-investigators.

An independent RAC at the KFSH&RC will serve as a Safety Monitoring Committee which includes faculty with expertise in various disciplines engaged in human subjects’ research from the hospital and research center, and also community members. Consultants with special expertise might be invited to assist from time to time with complex issues. The committee will undertake periodic reviews at the discretion of the Chair, and an expedited review is done for all serious unexpected adverse events (SUAEs), including death. The committee has the authority to suspend or halt recruitment if necessary. Refer to Supplementary File 2 for the form used to report SUAEs and death by the study investigators within 48 h of occurrence. All death cases reviewed so far have been due to the underlying disease, with participation in the trial not being a contributing factor. Any clinically significant worsening in a study participant’s condition based on clinical judgement compared to the baseline status at the time of randomization will be recorded as an AE in our progress report to be sent to the RAC by 4 June 2020 (the date specified originally upon IRB approval of the study protocol). This is applied whether or not the AE is considered to be related to the study treatment. In addition, this study is registered at the Saudi Food and Drug Authority (FDA), which provides independent input regarding the safety of interventions. Since this is an investigator-initiated, single-center, pilot, feasibility trial, periodic reviews are basically focused on monitoring safety. No formal interim analysis of efficacy will be undertaken due to possible small numbers that might preclude determination of a statistically significant difference in outcomes between the arms. No stopping rules or external independent Data Safety Monitoring Committee (DSMC) are specified. We believe the administration of sedative agents is standard of practice in the ICU to minimize a patient’s discomfort while on MV (see Supplementary Figure 1: KFSH&RC new sedation protocol for adult ICUs). Hence, the expected adverse effects will not exceed what is encountered during daily practice (e.g., benzodiazepine-associated delirium, opioid-induced constipation, hemodynamic instability associated with propofol and dexmedetomidine, ketamine-associated sympathetic stimulation “ tachycardia and increase in blood pressure,” and possible delirium). Nonetheless, an external and independent DSMC will be considered moving forward to a multisite RCT.

### Ancillary and post-trial care

As detailed in the Patient Information and Consent Form, any injury or complication occurring as a result of trial participation is to be reported to the study team, who will arrange all necessary medical treatment.

### Trial disseminationThe trial registration and dissemination information is as follows:

1. The trial was registered at ClinicalTrials.gov: Identifier NCT04075006 (registered on 30 August 2019), Saudi Food and Drug Authority: SCTR #19063002 (registered on 27 August 2019), and current controlled trials: ISRCTN14730035 (registered on 3 February 2020).
2. Trial results are to be presented at relevant scientific meetings and published in peer-reviewed journals.
3. The role of adjunct ketamine infusion as analgo-sedation is presented to the Saudi Critical Care Trials Group.
4. The trial will be publicized by ISRCTN via social media, and a trial blog will be available at the BMC website.

## Discussion

To the best of our knowledge, our pilot study is the first RCT that compares adjunct low-dose ketamine infusion to standard of care alone in critically ill patients. It is conducted in a mixed ICU cohort (medical, surgical, transplant, and oncology ICU settings), focused on patient-centered outcomes as a primary outcome (duration of MV), and addresses the fact that most patients in the ICU are sedated with a combination of drugs. Randomization, blinded study participants and study statistician, and adherence to the modified intention-to-treat principle will limit potential sources of bias. Another strength of this pilot study is the narrow randomization window (within 24 h post-intubation), which was chosen based on prior literature that showed early initiation of an intervention increases the ability of the intervention to influence the outcome, be more informative for clinicians, and have a greater power to detect an effect on important outcomes, such as duration of mechanical ventilation and long-term outcomes [16–18]. Moreover, we elected to record the vasopressor requirements, cumulative sedatives and analgesics, number of patients within RASS and pain score goals, and delirium incidence 48 h post-randomization to avoid the presence of confounders if measured > 48 h after ketamine infusion, similar to the study by Groetzinger et al. [11].

A concern was raised about the under-dosing of ketamine compared to ICU ketamine studies. Various dosing regimens of ketamine continuous infusion for sedation are described in the literature. A recent systematic review described the existing data regarding ketamine dosing for adjunct sedation in small cohorts of primarily neurologically injured patients [12]. The included studies describe dosing regimens up to 103.3 *μ* g/kg/min (6.2 mg/kg/h), which is substantially higher than the doses prescribed in our cohort. As most patients in the review had a neurological injury, sedatives were administered to maintain deep sedation, and often with background benzodiazepine infusion or even barbiturate anesthesia [12]. On the other hand, Groetzinger and colleagues used continuous infusion ketamine for adjunct sedation in a population of mechanically ventilated, critically ill adults targeting light sedation; ketamine was infused at a median starting dose of 1.6–4.2 *μ* g/kg/min (0.1–0.25 mg/kg/h) for a median of 2.8 days. The maximum doses of ketamine in individual patients experiencing adverse drug reactions (such as tachyarrhythmia) ranged from 2.08–20 *μ* g/kg/min (0.125 to 1.2 mg/kg/h), necessitating discontinuation of the infusion [11].

We aimed to describe our experience using ketamine as an adjunct low-dose sedative in an era that emphasizes light sedation in a complex, mechanically ventilated, critically ill population, specifically patients with medical illnesses, or following complicated surgical procedures. Therefore, we chose in our pilot study the dosing regimen based on the RCT cited by the 2018 PADIS guideline, which is comparable to the dosing regimen described in the study of Groetzinger et al. We believe this represents the safest dose as an adjunct analgo-sedative agent to decrease the risk of side effects, i.e., delirium, hallucinations, and tachycardia, through its sympathetic stimulation [1, 3, 11].

Limitations of our pilot study include the open-label design, as it has a process of multiple interventions related to the pain and sedation protocol. Therefore, the ICU treating team and the study investigators will know to which arm the study participants are randomized. Moreover, we will not collect data on other pain medications such as morphine and hydromorphone, as those medications are rarely used, per the KFSH&RC adult sedation protocol, compared to fentanyl. Another limitation is exclusion of patients in status asthmaticus or status epilepticus and patients placed on ECMO, as the dosing regimen of ketamine in those conditions is different than the regimen we used herein as an adjunct analgo-sedative agent. This may limit the external validity of this trial, although the population described in our cohort is relevant to many critically ill patients. Since we were interested in describing ketamine infusions as part of a light sedation strategy, we have excluded patients for whom the RASS goal is − 5, such as those receiving continuous infusion neuromuscular blockade. Additionally, the safety profile described in our cohort is not generalizable to higher doses of ketamine used to achieve deep sedation.

In conclusion, the findings of this pilot trial will contribute to a better understanding of adjunct low-dose ketamine infusion as an analgo-sedative agent and test the feasibility for a larger multicenter, randomized, double-blind, placebo-controlled trial with an adequate power to determine the effect of ketamine infusion as an analgo-sedative agent on clinical outcomes— mirroring other major sedation-related RCTs [18, 23, 28–30]. Future trials addressing cardiac assessment and hemodynamic metrics in a more protocolized way would be a great addition. For example, studies could assess metrics such as measurement of cardiac index (CI), stroke volume (SV), pulse pressure variation (PPV), and stroke volume variation (SVV) estimated by arterial pulse pressure waveform analysis (e.g., with the Vigileo monitor) at baseline (prerandomization) and at 24 and 48 h afterward (post-randomization), considering other potential confounders and adjunct interventions to evaluate whether these findings are direct consequences of ketamine or independent changes related to the severity of critical illness and the cumulative effect of adjunct interventions (i.e., antimicrobials, steroids, fluid administration, and blood products). Lastly, it would be optimal to assess some patient-reported outcomes for those who developed an emergence reaction or delirium. The active engagement of patients and family members has been highlighted recently in the ICU literature, and this would be a great addition to consider in a future multicenter trial through completing an ICU diary or a survey 28 days post-ICU discharge among ICU survivors [31].

## Trial status

As of 10 October 2019, a total of 16 patients have been enrolled. We expect to complete the recruitment by 31 December 2020. The trial was first approved on 13 July 2019 (Protocol V1) and opened to recruitment on 1 September 2019 (Protocol V1). The protocol was amended on 3 September 2019 (Protocol V2) requesting initial waiver of consent, as we faced difficulty with the patient enrollment and consenting prior to randomization due to inability to reach the legal surrogate (not answering the phone for verbal consent) or the emotional factor with the legal surrogate, especially during the first 24 h post-intubation and ICU admission. However, our Research Ethics Committee mandated the informed consent prior to randomization. Another protocol amendment (Protocol V3) on 24 February 2020 reflected the clarifications that were made in the revised version.

## Supplementary information

**Additional file 1:Supplementary Table 1.** Previous ketamine trials in the ICU setting. **Supplementary Figure 1.** KFSH&RC new sedation protocol for adult intensive care units. **Supplementary File 1.** Standard Protocol Items: Recommendations for Interventional Trials (SPIRIT) checklist. **Supplementary Table 2.** Adjunct low-dose ketamine infusion vs standard of care in mechanically ventilated critically ill patients at a tertiary Saudi hospital (ATTAINMENT trial) SPIRIT protocol summary. **Supplementary File 2.** Serious Unexpected Adverse Event (SUAE) Report Form.

## Data Availability

All data in the study protocol are included in this published article and its supplementary information files.

## Abbreviations

AE: Adverse event
APACHE II: Acute Physiology and Chronic Health Evaluation II
CI: Cardiac index
CRC: Clinical Research Committee
CAM-ICU: Confusion Assessment Method for the ICU
DSMC: Data Safety Monitoring Committee
DNR: Do Not Resuscitate
ECMO: Extracorporeal membrane oxygenation
HR: Heart rate
ICU: Intensive care unit
IQR: Interquartile range
IRB: Institutional Review Board
KFSH&RC: King Faisal Specialist Hospital and Research Center
LOS: Length of stay
MAP: Mean arterial pressure
MV: Mechanical ventilation
CPIS: Modified Clinical Pulmonary Infection Score
ORA: Office of Research Affairs
PADIS: Pain, Agitation/sedation, Delirium, Immobility, and Sleep disruption
RCT: Randomized controlled trial
PF ratio: Ratio of the partial pressure of arterial oxygen to the fraction of inspired oxygen
REC: Research Ethics Committee
REDCap: Research Electronic Data Capture
RASS: Richmond Agitation-Sedation Scale
SOFA: Sequential Organ Failure Assessment
SUAE: Serious unexpected adverse event
SAT: Spontaneous awakening trial
SBT: Spontaneous breathing trial
SV: Stroke volume
SVV: Stroke volume variation
SBP: Systolic blood pressure
VAP: Ventilator-associated pneumonia
