# Supplementary material for: Adjunct low-dose ketamine infusion vs standard of care in mechanically ventilated critically ill patients at a Tertiary Saudi Hospital (ATTAINMENT Trial): study protocol for a randomized, prospective, pilot, feasibility trial
Source: Trials. 2020 Mar 20;21:288. doi: 10.1186/s13063-020-4216-4 (PMC7085173; doi:10.1186/s13063-020-4216-4)
Supplement: Supplementary file 1 — Additional file 1:Supplementary Table 1. Previous ketamine trials in the ICU setting. Supplementary Figure 1. KFSH&RC new sedation protocol for adult intensive care units. Supplementary File 1. Standard Protocol Items: Recommendations for Interventional Trials (SPIRIT) checklist. Supplementary Table 2. Adjunct low-dose ketamine infusion vs standard of care in mechanically ventilated critically ill patients at a tertiary Saudi hospital (ATTAINMENT trial) SPIRIT protocol summary. Supplementary File 2. Serious Unexpected Adverse Event (SUAE) Report Form. [file 13063_2020_4216_MOESM1_ESM.pdf]

Additional/ Supplementary Files

Supplementary Table 1: Previous Ketamine Trials in the ICU setting

Ref.	Design	Drug Regimens	Patients	Results/Comments
Whitman et al. [2]	Retrospective chart review	Median duration of ketamine therapy was 1.57 days. Median minimum and maximum rates were 0.24 mg/kg/h and 0.83 mg/kg/h	12 MICU on MV	Ketamine had to be discontinued in 2 patients because of hypertension and tachycardia.<br>Five patients had their sedative and analgesic doses reduced.<br>**Conclusion**: continuous-infusion of ketamine can be safely used for adjunctive sedation and analgesia in mechanically ventilated patients in the medical ICU.
Guillou [3]	Randomized, double-blinded study	Ketamine versus placebo<br>Dose: 0.5 mg/kg bolus, 1-2 µg/kg/min (0.06-0.12 mg/ kg/h) for 48 hours	93 Surgery<br>All patients received morphine via PCA	↓ Cumulative morphine consumption (22 mg).<br>No difference in pain and Ramsay sedation scores.
Moitra et al. [4]	Case series	Doses of ketamine infusion ranged from 0.5 to 4 µg/kg/min. A low-dose ketamine infusion was used to reduce agitation in a patient requiring high doses of sedatives and analgesics	4 critically ill patients with varying complications related to prolonged critical illness	Low-dose ketamine infusion was used to reduce agitation in a patient requiring high doses of sedatives and analgesics. In a second patient, ketamine improved depression and anxiety symptoms. In a third patient, ketamine may have facilitated liberation from mechanical ventilation. In a fourth patient, ketamine was used for palliation to avoid lethargy.<br>**Conclusion** Ketamine may be considered to assist in decreasing agitation, managing pain, facilitating opioid and benzodiazepine withdrawal, preventing respiratory depression, and potentially managing depression and anxiety in chronic critically ill patients.

Ref.	Design	Drug Regimens	Patients	Results/Comments
Garber et al. [5]	Retrospective two-center intra-patient comparison study, including mechanically ventilated patients	Median ketamine infusion starting dose was 5.0 µg/kg/minute (IQR 2.6–5.0 µg/kg/min) and median duration was 90.5 hours (IQR 55.9–148.0 h).	104 adult ICU patients	A 20% (IQR - 63.6 to 0.0, p<0.001) relative reduction in total analgesic- sedative infusion pharmacotherapy was achieved at 24 hours after ketamine initiation. Analgesic and sedative infusion doses decreased at 24 hours (fentanyl: pre, 175 μg/h [IQR 100–200 μg/h] vs post, 125 μg/h [IQR 50–200 μg/h], p<0.001; propofol: pre, 42.5 μg/kg/min [IQR 20.0–60.0 μg/kg/ min] vs post, 20.0 μg/kg/min [IQR 3.8–31.3 µg/kg/min], p<0.001).<br><br>Median percent time within the RASS goal improved after ketamine initiation (pre, 7.1% [0–40%] vs post, 25% [0–66.7%], p=0.005).<br><br>Independent factors associated with ketamine response included a lower body mass index, higher starting dose of ketamine, lower severity of illness, and the need for multiple concomitant analgesic-sedative infusions before ketamine initiation.
Pruskowski et al. [6]	Single-center, retrospective chart review over a 19- month period	Ketamine infusion was 0.64 ± 0.39 mg/kg/h for the first 24 hours<br><br>Between 24 and 48 hours, this rate was 0.81 ± 0.56 mg/kg/h<br><br>Between 48 and 72 hours, rate of infusion was 0.94 ± 0.62 mg/kg/h	36 critically ill, MV, trauma patients<br><br>81% male<br><br>58% blunt trauma<br><br>11% CAM-ICU positive before ketamine	↓in the amount of opioids and propofol used and an ↑in the amount of ziprasidone and dexmedetomidine needed to achieve the Richmond Agitation Score goal.<br><br>No difference in the proportion of time that patients took to achieve the sedation goal after ketamine, duration of MV, ICU or hospital LOS.
Bourgoin et al. [7]	Prospective, randomized, double-blind study	Ketamine versus sufentanil<br><br>All patients received	25 traumatic brain injury patients	No difference in intracranial pressure (ICP) or cerebral perfusion pressure (CPP).

Ref.	Design	Drug Regimens	Patients	Results/Comments
		midazolam 82 mg/kg/min (5 mg/kg/h) for 6 days		
Shurtleff et al. [8]	Retrospective cohort study conducted at an academic medical center in the United States	2 groups based on the sedative regimen used: ketamine-based or non-ketamine-based<br>Ketamine starting infusion rate was 5 μg/kg/min. This could be titrated using 5 μg/kg/min every 5 minutes up to a maximum of 25 μg/kg/min.	79 patients; 39 received ketamine-based while 40 received non-ketamine-based sedation	Number of days alive without delirium or coma was 6 days (IQR: 2-9 days) with ketamine and 4 days (IQR: 3-7 days) with non-ketamine (P = 0.351).<br>Delirium occurred in 29 (74%) of the 39 patients treated with ketamine and 34 (85%) of the 40 patients treated with non-ketamine (P = 0.274).<br>Coma occurred in 16 (41%) of the 39 patients treated with ketamine and 6 (15%) of the 40 patients treated with non-ketamine (P = 0.013).<br>Median ventilator-free days were 13 days (IQR: 0-23 days) with ketamine and 21 days (0-25 days) with non-ketamine (P = 0.229).<br>**Conclusions**: Sustained ketamine-based sedation in mechanically ventilated patients may be associated with a higher rate of observed coma but similar delirium- and coma-free days compared to non-ketamine-based regimens.
Buchheit et al. [9]	Retrospective review	Low-dose ketamine continuous infusion (1-5 μg/kg/min) for adjunctive pain. Control treatment duration was ~2 days	40 mechanically ventilated adult patients in the surgical ICU, if they met an ICU safety screen for a spontaneous breathing trial (SBT), implying extubation readiness pending SBT results	Prior to ketamine, most patients received volume-controlled or pressure-supported ventilation with a median duration of 2.05 days (IQR: 1.38-3.61).<br>Median time from initiation of ketamine to extubation was 1.44 days (IQR: 0.58-2.66). For primary outcome, there was a significant difference in the slope of morphine equivalent changes pre- and post-ketamine initiation (P < 0.001).<br>↓in vasopressor requirements (phenylephrine equivalent 70 vs 40 mg/h; P = 0.019).

Ref.	Design	Drug Regimens	Patients	Results/Comments
Perbet et al. [10]	RCT double-blinded	Divided into ketamine (2 mg/kg/h) or placebo group	Patients were admitted in a general ICU of a university hospital and were undergoing mechanical ventilation (n = 162) with a nurse-driven sedation protocol	Daily consumption of remifentanil (7.9 +/- 1.0 vs. 9.3 +/- 1.0 μg/kg/h, P = 0.548) and the ↑in remifentanil doses required for equianalgesia (0.107 +/- 0.17 and 0.11 +/- 0.18 μg/kg/min, P = 0.78) were not different between ketamine and control groups.<br><br>Duration of delirium was lower in the ketamine group than placebo group (5.3 +/- 4.7 vs. 2.8 +/- 3 days, P = 0.005).<br><br>Mortality rates, ventilator-free days, and ICU length of stay (LOS) were not statistically different between the groups.
Groetzinger et al. [11]	Retrospective review	Ketamine was infused at a median dosage of 0.41 mg/kg/h (range 0.04-2.5 mg/kg/h) for up to 14.7 days (median 2.8 days).	N= 91 mechanically ventilated patients receiving continuous ketamine infusion between January 2012 and April 2016<br><br>59% MICU<br><br>20% transplant ICU<br><br>15% trauma ICU	63% patients' concomitant sedatives were reduced or discontinued, without the initiation of an additional sedative within 24 hours of initiating ketamine.<br><br>Propofol was most commonly discontinued (16 patients, 36%), followed by benzodiazepines (12 patients, 27%).<br><br>There was an ↑in the number of SAS scores documented in the 24-hour period after ketamine initiation compared to the immediate 24 hours before initiation (61% vs 55%, p=0.001).<br><br>Patients were less frequently agitated, defined as SAS >4, after initiation of ketamine (27% vs 33%, p=0.005).<br><br>7.7% of patients required discontinuation of ketamine infusion for adverse drug reactions.<br><br>There were no significant changes in hemodynamic variables after initiation of ketamine.

Ref.	Design	Drug Regimens	Patients	Results/Comments
				**Conclusions**:<br>Continuous ketamine infusion as an adjunct light sedation was well-tolerated in a cohort of critically ill adults, with an acceptable safety profile. Prospective studies of ketamine infusion are warranted to further establish its efficacy as a sedative in this population.

Supplementary Figure 1: KFSH&RC New Sedation Protocol for Adult Intensive Care Units

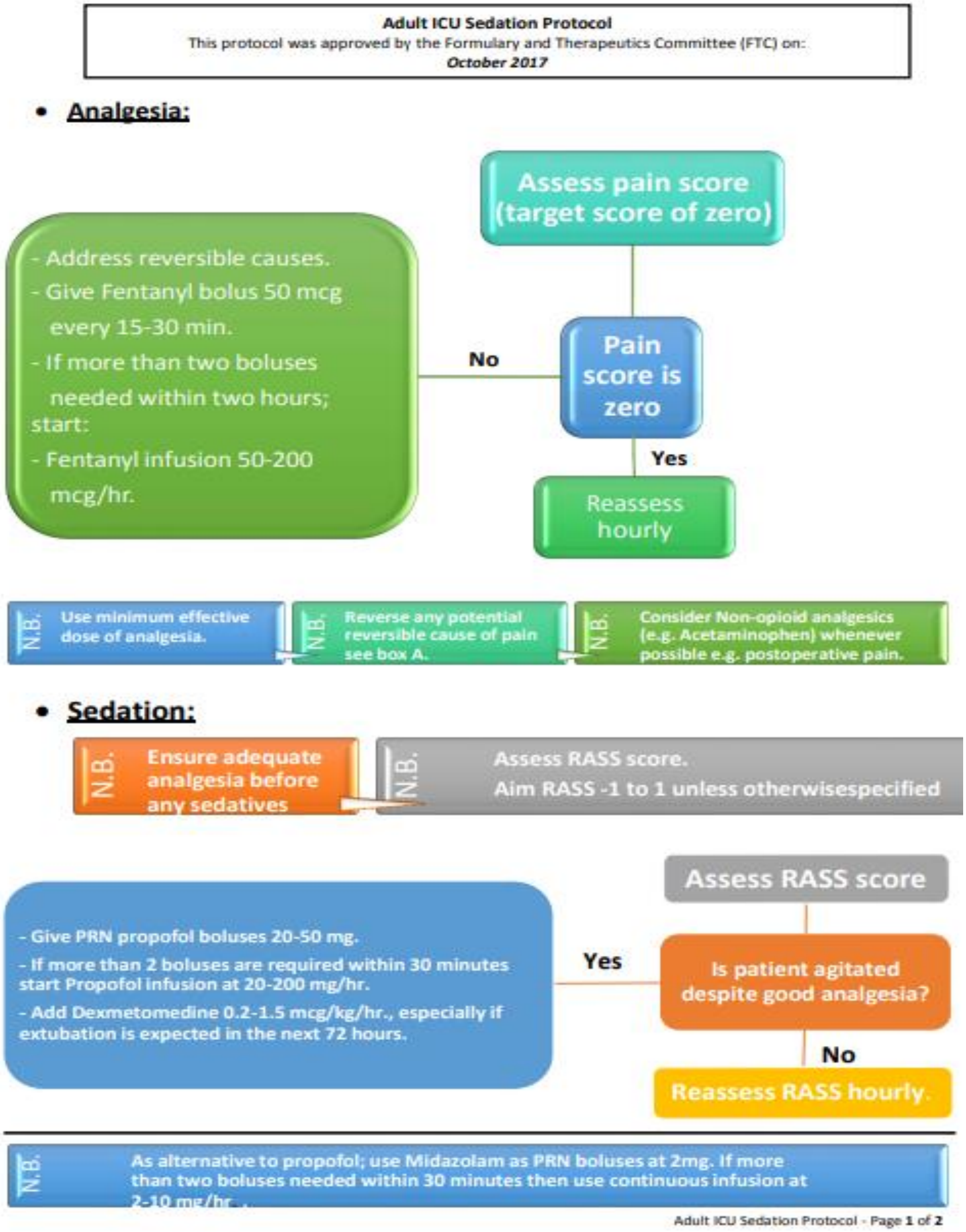

N.B.	Use alternative to propofol (e.g. Midazolam) if the patient is in severe shock requiring norepinephrine more than 0.5mcg/kg/min. Midazolam may be used if extubation is anticipated within 24 hours.
N.B.	Daily Spontaneous awakening trial/sedation Vacation at 0900 is mandatory unless otherwise specified, stop all sedatives and allow patient to wake up see box B. If the patient wakes up, assess for Spontaneous breathing trial.
N.B	Ketamine infusion is a valid choice especially in patients with severe bronchospasm (Form B is required).
N.B	If Muscle relaxants are used (nfusion or boluses), then patient MUST be on BIS monitoring with a target BIS 40-50.

- **<u>Delirium:</u>**

First	Second	Third
CAM-ICU must be documented once /shift, if hyperactive delirium then inform MD	Try non-pharmacological solutions such as re-orientation of the patient, relatives attendance see box C.	Consider using Haloperidol, Quetiapine.

Appendix:

Box A:

Reversible causes of pain:

- Medication, drugs/ETOH withdrawal.
- Ischemia/ Hypoxia
- Medical emergencies (pneumothorax).
- Malposition of ET tube.
- Urinary retention/ constipation

Box B:

Contraindications to spontaneous awakening trial:

- Paralyzed patient.
- Haemodynamic instability.
- FiO2> 60%.
- PEEP > 10 cmH2O.
- Non-conventional mode of ventilation e.g. APRV, HFOV.
- Traumatic brain injury.
- Open abdominal wound.
- Recent <3 weeks myocardial infarction.

Box C:

Non-pharmacological treatments of delirium:

- Positioning.
- Eliminate irritating physical stimuli e.g. Movement of the ETT.
- Dimming the Lights.
- Reducing the noise level.
- Family support.
- Sleep promotion.
- Calm environment

STANDARD PROTOCOL ITEMS: RECOMMENDATIONS FOR INTERVENTIONAL TRIALS

Supplementary File 1 Standard Protocol Items: Recommendations for Interventional Trials (SPIRIT) checklist

Recommended items to address in a clinical trial protocol and related documents*

Section/item	Item No	Description	Addressed on page number
**Administrative information**			
Title	1	Descriptive title identifying the study design, population, interventions, and, if applicable, trial acronym	_______1____
Trial registration	2a	Trial identifier and registry name. If not yet registered, name of intended registry	_______3_____
	2b	All items from the World Health Organization Trial Registration Data Set	_______3_______
Protocol version	3	Date and version identifier	_____3, 25_____
Funding	4	Sources and types of financial, material, and other support	_______28______
Roles and responsibilities	5a	Names, affiliations, and roles of protocol contributors	_____27-28____
	5b	Name and contact information for the trial sponsor	_______28_______
	5c	Role of study sponsor and funders, if any, in study design; collection, management, analysis, and interpretation of data; writing of the report; and the decision to submit the report for publication, including whether they will have ultimate authority over any of these activities	____ 28_______
	5d	Composition, roles, and responsibilities of the coordinating centre, steering committee, endpoint adjudication committee, data management team, and other individuals or groups overseeing the trial, if applicable (see Item 21a for data monitoring committee)	_15-16, 19-21___

Introduction

Background and rationale	6a	Description of research question and justification for undertaking the trial, including summary of relevant studies (published and unpublished) examining benefits and harms for each intervention	___ 4 - 8____
	6b	Explanation for choice of comparators	____ 4 - 8____
Objectives	7	Specific objectives or hypotheses	____ 8 -9_____
Trial design	8	Description of trial design including type of trial (eg, parallel group, crossover, factorial, single group), allocation ratio, and framework (eg, superiority, equivalence, noninferiority, exploratory)	_____ 9______

Methods: Participants, interventions, and outcomes

Study setting	9	Description of study settings (eg, community clinic, academic hospital) and list of countries where data will be collected. Reference to where list of study sites can be obtained	______9________
Eligibility criteria	10	Inclusion and exclusion criteria for participants. If applicable, eligibility criteria for study centres and individuals who will perform the interventions (eg, surgeons, psychotherapists)	_____9-10_____
Interventions	11a	Interventions for each group with sufficient detail to allow replication, including how and when they will be administered	_____ 11_____
	11b	Criteria for discontinuing or modifying allocated interventions for a given trial participant (eg, drug dose change in response to harms, participant request, or improving/worsening disease)	______11-12_____
	11c	Strategies to improve adherence to intervention protocols, and any procedures for monitoring adherence (eg, drug tablet return, laboratory tests)	_____15-16_____
	11d	Relevant concomitant care and interventions that are permitted or prohibited during the trial	______14_______
Outcomes	12	Primary, secondary, and other outcomes, including the specific measurement variable (eg, systolic blood pressure), analysis metric (eg, change from baseline, final value, time to event), method of aggregation (eg, median, proportion), and time point for each outcome. Explanation of the clinical relevance of chosen efficacy and harm outcomes is strongly recommended	_____14-15____

Participant timeline	13	Time schedule of enrolment, interventions (including any run-ins and washouts), assessments, and visits for participants. A schematic diagram is highly recommended (see Figure)	_______13________
Sample size	14	Estimated number of participants needed to achieve study objectives and how it was determined, including clinical and statistical assumptions supporting any sample size calculations	______17-19______
Recruitment	15	Strategies for achieving adequate participant enrolment to reach target sample size	_______19________

Methods: Assignment of interventions (for controlled trials)

Allocation:

Sequence generation	16a	Method of generating the allocation sequence (eg, computer-generated random numbers), and list of any factors for stratification. To reduce predictability of a random sequence, details of any planned restriction (eg, blocking) should be provided in a separate document that is unavailable to those who enrol participants or assign interventions	________12 _____
Allocation concealment mechanism	16b	Mechanism of implementing the allocation sequence (eg, central telephone; sequentially numbered, opaque, sealed envelopes), describing any steps to conceal the sequence until interventions are assigned	________12 ____
Implementation	16c	Who will generate the allocation sequence, who will enrol participants, and who will assign participants to interventions	________12 _______
Blinding (masking)	17a	Who will be blinded after assignment to interventions (eg, trial participants, care providers, outcome assessors, data analysts), and how	________13_______
	17b	If blinded, circumstances under which unblinding is permissible, and procedure for revealing a participant's allocated intervention during the trial	________13_______

Methods: Data collection, management, and analysis

Data collection methods	18a	Plans for assessment and collection of outcome, baseline, and other trial data, including any related processes to promote data quality (eg, duplicate measurements, training of assessors) and a description of study instruments (eg, questionnaires, laboratory tests) along with their reliability and validity, if known. Reference to where data collection forms can be found, if not in the protocol	_______15-17_____

	18b	Plans to promote participant retention and complete follow-up, including list of any outcome data to be collected for participants who discontinue or deviate from intervention protocols	___15-17 and Table 1: Intervention Stopping Rule and Protocol Deviation [action regarding study procedure]
Data management	19	Plans for data entry, coding, security, and storage, including any related processes to promote data quality (eg, double data entry; range checks for data values). Reference to where details of data management procedures can be found, if not in the protocol	_____15-16_____
Statistical methods	20a	Statistical methods for analysing primary and secondary outcomes. Reference to where other details of the statistical analysis plan can be found, if not in the protocol	_____17-19_______
	20b	Methods for any additional analyses (eg, subgroup and adjusted analyses)	_____18-19_____
	20c	Definition of analysis population relating to protocol non-adherence (eg, as randomised analysis), and any statistical methods to handle missing data (eg, multiple imputation)	_____ 19____
**Methods: Monitoring**			
Data monitoring	21a	Composition of data monitoring committee (DMC); summary of its role and reporting structure; statement of whether it is independent from the sponsor and competing interests; and reference to where further details about its charter can be found, if not in the protocol. Alternatively, an explanation of why a DMC is not needed	_____19-21_____
	21b	Description of any interim analyses and stopping guidelines, including who will have access to these interim results and make the final decision to terminate the trial	_____19- 21____
Harms	22	Plans for collecting, assessing, reporting, and managing solicited and spontaneously reported adverse events and other unintended effects of trial interventions or trial conduct	_____19-21___
Auditing	23	Frequency and procedures for auditing trial conduct, if any, and whether the process will be independent from investigators and the sponsor	_____16 ________

Ethics and dissemination

Research ethics approval	24	Plans for seeking research ethics committee/institutional review board (REC/IRB) approval	_____10_____
Protocol amendments	25	Plans for communicating important protocol modifications (eg, changes to eligibility criteria, outcomes, analyses) to relevant parties (eg, investigators, REC/IRBs, trial participants, trial registries, journals, regulators)	_____25______
Consent or assent	26a	Who will obtain informed consent or assent from potential trial participants or authorised surrogates, and how (see Item 32)	____ 10-11______
	26b	Additional consent provisions for collection and use of participant data and biological specimens in ancillary studies, if applicable	_____N/A _______
Confidentiality	27	How personal information about potential and enrolled participants will be collected, shared, and maintained in order to protect confidentiality before, during, and after the trial	_____15-16____
Declaration of interests	28	Financial and other competing interests for principal investigators for the overall trial and each study site	______28_______
Access to data	29	Statement of who will have access to the final trial dataset, and disclosure of contractual agreements that limit such access for investigators	____19_, 27-28_____
Ancillary and post-trial care	30	Provisions, if any, for ancillary and post-trial care, and for compensation to those who suffer harm from trial participation	______21_________
Dissemination policy	31a	Plans for investigators and sponsor to communicate trial results to participants, healthcare professionals, the public, and other relevant groups (eg, via publication, reporting in results databases, or other data sharing arrangements), including any publication restrictions	______21-22_______
	31b	Authorship eligibility guidelines and any intended use of professional writers	_______ 28 ______
	31c	Plans, if any, for granting public access to the full protocol, participant-level dataset, and statistical code	_______ N/A _____

Appendices

Informed consent materials	32	Model consent form and other related documentation given to participants and authorised surrogates	________ N/A ______
Biological specimens	33	Plans for collection, laboratory evaluation, and storage of biological specimens for genetic or molecular analysis in the current trial and for future use in ancillary studies, if applicable	________ N/A ______

*It is strongly recommended that this checklist be read in conjunction with the SPIRIT 2013 Explanation & Elaboration for important clarification on the items. Amendments to the protocol should be tracked and dated. The SPIRIT checklist is copyrighted by the SPIRIT Group under the Creative Commons "Attribution-NonCommercial-NoDerivs 3.0 Unported" license.

Supplementary Table 2 Adjunct Low-Dose Ketamine Infusion versus Standard of Care in Mechanically Ventilated Critically Ill Patients at a Tertiary Saudi Hospital (ATTAINMENT) Trial Standard Protocol Items: Recommendations for Interventional Trials (SPIRIT) protocol summary

Scientific title	Adjunct Low-Dose Ketamine Infusion versus Standard of Care in Mechanically Ventilated Critically Ill Patients at a Tertiary Saudi Hospital (ATTAINMENT Trial): A Study Protocol for a Randomized, Prospective, Pilot, Feasibility Trial
Short title	Use of adjunct continuous ketamine infusion in mechanically ventilated patients
Health condition	Mechanically ventilated adult critical illness
Ethics	Approved July 13, 2019, Research Ethics Committee (REC) and Clinical Research Committee (CRC) at King Faisal Specialist Hospital and Research Center (KFSHRC) with a Research Advisory Council (RAC) number 2191 187 (MBC-03, PO Box 3354, Riyadh 11211, Saudi Arabia; +966114424528; aomar@kfshrc.edu.sa
Protocol version	Version 3, First approved on July 13, 2019
Funding	This trial is investigator-initiated and all study authors are employees at KFSH&RC which has not provided any research grant for this particular project. All authors are expected to volunteer their time and use local resources to conduct the study.<br>Industry:<br>Nil
Primary sponsor	Investigator-initiated and -driven study.<br>Research Advisory Council at King Faisal Specialist Hospital and Research Center (KFSH&RC), Riyadh , Saudi Arabia. Principle investigators and study contact:<br>Dr Mohammed Bawazeer mbawazeer@kfshrc.edu.sa<br>Dr Marwa Amer mamer@kfshrc.edu.sa<br>King Faisal Specialist Hospital & Research Centre, P.O Box 3354, Riyadh, Saudi Arabia, 11211
Background	Ketamine is used to produce sedation and relieve pain to minimize discomfort while a breathing tube and a machine (ventilator) used in the ICU. Several publications have shown that a low-dose ketamine in combination to opioids has been used to relieve acute pain after surgery. Ketamine has a favorable characteristics including bronchodilation, increase in blood pressure, does not cause constipation, maintain respiratory reflexes make it an especially viable alternative for patients with unstable respiratory and hemodynamic function. However, the majority of these trials are conducted in a surgical ICU setting, retrospective in nature or randomized controlled clinical trials focusing on comparison of ketamine to placebo or two study drugs (e.g. ketamine versus opioid), despite the fact that most ICU patients are sedated with a combination of drugs.<br>The aim of this study is to assess weather adjunct ketamine can help to shorten the time of being in breathing tube and ventilator (duration of mechanical ventilation) compared to standard of care (without ketamine). Together these data will be used to inform the design of a large multicentre trial to assess clinical outcomes
Hypothesis	low-dose ketamine infusion will reduce the duration of mechanical ventilation (MV) with an acceptable safety profile compared to standard of care

Study aims	**Primary**: To study the feasibility and effect of adjunct low-dose ketamine infusion on MV duration compared to the standard of care alone in critically ill patients.<br>**Secondary**: To study the effect of adjunct low-dose ketamine infusion on the following:<br>1- The cumulative dose of pain and sedative medications.<br>2- The incidence of dexmedetomidine use post randomization.<br>3- The number of patients are in RASS and pain score goal.<br>4- The hemodynamic status in terms of vasopressor therapy requirement.<br>5- ICU and hospital length of stay (LOS).<br>6- Tracheostomy, unplanned extubation, and re-intubation rates.<br>7- The incidence of delirium and rate of positive Confusion Assessment Method in Intensive Care Unit (CAM-ICU).<br>8- The rate of antipsychotic use for ICU-acquired delirium.<br>9- The rate of hypersalivation and frequent suctioning.<br>10- The rate of using physical restraint.<br>11- Mortality rate at 28 days.
Study design	• Prospective, randomized, single center, open-label active controlled parallel group pilot feasibility, phase 3 study<br>• Interventional
Setting	KFSHRC adult ICUs (medical , surgical, and transplant/oncology), Riyadh, Saudi Arabia
Inclusion and exclusion criteria	**Inclusion criteria:**<br>• Adult ICU patients (> 14 years old) on MV admitted to one of the following ICUs: medical, surgical, or transplant/oncology ICU.<br>• Intubated within the previous 24 hours.<br>• Expected to require MV for more than 24 hours.<br>• Expected to be on the KFSH&RC ICU sedation and pain protocol.<br>• No objection from the ICU attending MD for enrollment.<br><br>**Exclusion criteria:**<br>• Patients with a history of dementia or psychiatric disorders or on any antipsychotics or antidepressants medications at home.<br>• Pregnancy.<br>• Age <14 years old.<br>• Expected to need MV < 24 h.<br>• Known hypersensitivity to ketamine.<br>• Patients with expected targeted RASS of -5 such as patients on continuous infusion neuromuscular blockade.<br>• Patients on dexmedetomidine as the primary sedative prior to randomization.<br>• Patients with cardiogenic shock, acute decompensated heart failure, or myocardial infarction.<br>• History of end-stage liver failure (child Pugh score C).<br>• Proven or suspected primary neurological injury (traumatic brain injury, ischemic stroke, intracranial hemorrhage, spinal cord injury, anoxic brain injury, brain edema).<br>• Patients with persistent heart rate (HR) > 150 bpm or systolic blood pressure (SBP) >180 mmHg.<br>• Patients identified as do-not-resuscitate (DNR) or expected to die within 24 hours.<br>• Patients on extracorporeal membrane oxygenation (ECMO).<br>• Patients with refractory status epilepticus who are receiving ketamine infusion.<br>• Proven or suspected status of asthmaticus.

Intervention	*Intervention arm*: Ketamine Group<br>Adjunct low dose continuous infusion ketamine in addition to the standard of care. Ketamine will be given at a fixed infusion rate of 0.12 mg/kg/hr (2 µg/kg/min) in the first 24 hours followed by 0.06 mg/kg/hr (1 µg/kg/min) in the second 24 hours, then discontinued<br><br>*Compotator arm* : Standard of care in the ICU including propofol and/or fentanyl and/or midazolam according to KFSHRC sedation and analgesia protocol.
Primary outcome measure	• Median duration of mechanical ventilation [from Intubation to extubation date and off mechanical ventilation or until ICU discharge, death, or 28 days post randomization whichever occurs first]<br>• Median ventilator-free days to day 28
Secondary outcome measures	**Clinical outcome measures:**<br>• Proportion and median cumulative dose of pain and sedative medications in the first 48 hours after randomization.<br>• Proportion of patients started on dexmedetomidine 48 hours after randomization.<br>• Proportion of patients achieving the RASS goal and pain score goal within the first 48 hours after randomization.<br>• Proportion of and median vasopressor requirements in the first 48 hours after randomization.<br>• Median change in mean arterial pressure (MAP) and Heart rate (HR) in the first 48 hours after randomization<br>• Median ICU and hospital LOS<br>• Proportion of tracheostomy, unplanned extubation (self extubation), and re-intubation within 28 days post randomization.<br>• Proportion of patients starting on antipsychotics and positive CAM-ICU score to assess the incidence of delirium 48 hours after randomization.<br>• Proportion of patient with frequent suctioning in the first 48 hours after randomization<br>• Proportion of physical restraint 48 hours after randomization.<br>• Mortality rate at the time of hospital discharge or 28 days after randomization, whichever comes first.<br>**Feasibility outcome measures:**<br>• Proportion of screened patients.<br>• Proportion of eligible patients enrolled.<br>• Enrolment rate (i.e. number of enrolments per month).<br>• Protocol compliance.
Sample size	40 per arm/80 total
Randomisation	Eligible participants in whom a consent process has been commenced will be randomised to either the Adjunct low dose continuous infusion ketamine (intervention arm) or the standard of care in the ICU including propofol and/or fentanyl and/or midazolam (control arm)
Randomisation procedure	• 1:1 randomisation and no stratification will be performed<br>• Patients will be randomly allocated to one of the two study groups based on a computer-generated randomization table, created by an independent biostatistician<br>• Our initial screening for patient eligibility is done by bedside ICU nurses who are blinded to treatment assignment. To further ensure allocation concealment, access to the randomization table will be restricted to a Pharmacist (third party and not part of the study) to whom principal investigators refers at distance to know the assigned

	treatment (by telephone). The study investigators and study participants during recruitment and consenting process will be blinded to treatment assignment. Once consenting process complete, principal investigators will contact the Pharmacist (third party) for patient allocation and initiation of the trial intervention. Group allocation will be concealed until after randomization
Blinding	• The study investigators and study participants during recruitment and consenting process will be blinded to treatment assignment.<br>• Once the trial intervention start, the treating team and study investigators will not be blind to the trial intervention for practical and safety purposes [open label]<br>• The study statistician is blinded to the treatment allocation and study investigators will remain blinded to the results until the conclusion of the study.
Data collection methods	• Data will be collected in the KFSH&RC Research Electronic Data Capture (REDCap) platform. Each subject will be given a unique subject ID number (database numbers and all identifiers will be removed). A subject ID key will be used to match the subjects' Medical Record Number and will be kept in a password-protected file that is accessible to the principal investigators. Access to the RedCap data will be limited to the principal investigators and coinvestigators involved in data collection only. Access to REDCap requires authentication (username and password) for secure maintenance of the data<br>• Periodic internal audits of data entry accuracy and compliance will be done by the principle investigators
Data collected	• **Baseline demographics:** age, gender, weight, mode of mechanical ventilation at baseline, percentage of renal replacement therapy at baseline, lactate level at baseline, and severity of illness as estimated by Sequential Organ Failure Assessment (SOFA) score and Acute Physiology and Chronic Health Evaluation (APACHE II) score, ICU type, comorbidities, baseline analgesics, sedatives, vasopressor requirements, and Pre-Deliric Delirium Risk Score, which is a delirium prediction model specifically designed for adult critical care patients 24 hours after ICU admission. It will be used to predict the factors that may influence delirium risk prior to randomization.<br>• We will also collect RASS, pain scores, and CAM-ICU scores at baseline, 24 and 48 hours post-randomization. Patients with a RASS of -3 or lower will be excluded from CAM-ICU assessment as they cannot participate in the exam.<br>• Modified Clinical Pulmonary Infection Score (CPIS).<br>• We will also record the proportion of eligible participants enrolled, rates of recruitment, protocol deviations and adverse events.
Statistical analysis	• Modified intention-to-treat principle, and will be comprised of data from all patients who undergo randomization, with the exception of those who withdraw consent, have an unknown primary outcome, and are identified as ineligible after randomization.<br>• The study statistician is blinded to the treatment allocation and study investigators will remain blinded to the results until the conclusion of the study.
Data and safety monitoring	• Principle investigators will meet weekly, review enrolment rates and coordinate the study in general . This will allow to identify any protocol deviations and provide opportunity for feedback to the other co-investigators. There will be periodic internal audits of data accuracy and compliance<br>• Serious unexpected adverse events (SUAE) including death will be reported to Research Advisory Council at the KFSH&RC within 48 hours of occurrence<br>• Research Advisory Council at the KFSH&RC will serve as Safety Monitoring Committee and review all SUAE and have the authority to suspend or halt recruitment if necessary

	• Periodic data review by Research Advisory Council at the KFSHRC and recommendations to PI in event of study conduct or SUAE issues
Ethics and governance	• The study will be conducted in accordance with Good Clinical Practice<br>• Trial is approved by the Institutional Review Board (IRB) of the KFSH&RC<br>• The study protocol as well as the informed consent have been approved by Research Ethics Committee (REC) and Clinical Research Committee (CRC) at KFSH&RC with a Research Advisory Council (RAC) number 2191 187
Consent	• Where possible, prospective informed consent will be obtained<br>• Due to the time-critical nature of the condition, approval in place for verbal consent to randomise and initiate care followed by formal written consent process<br>• Some participants will lack capacity so consent will be sought from next of kin
Confidentiality	• All data will be de-identified<br>• Data (paper and electronic) will be stored securely
Dissemination	• Trial was registered at ClinicalTrials.gov Identifier: NCT04075006 (registered on 30 August, 2019), Saudi Food and Drug Authority: SCTR #19063002 (registered on 27 August, 2019), and current controlled trials: ISRCTN14730035 (registered on 3 February 2020).<br>• Trial results are to be presented at relevant scientific meetings and published in peer-reviewed journal.<br>• The role of adjunct Ketamine Infusion as analgosedation is presented at the Saudi Critical Care Trial Group.<br>• The trial will be publicized by ISRCTN via social media, and a trial blog will be available at BMC website.
Study status	• Opened to recruitment on September 1, 2019 (Protocol V1)<br>• Protocol amended on September 3, 2019 (Protocol V2) requesting for initial waiver of consent as we faced difficulty with the patient enrollment and consenting prior to randomization due to inability to reach the legal surrogate (not answering the phone for verbal consent) or the emotional factor with legal surrogate especially the first 24-hour post intubation and ICU admission ,however, our Research Ethics Committee mandated the informed consent prior to randomization.<br>• Another protocol amended (Protocol V3) on February 24, 2020 to reflect the clarifications that were made in the revised version<br>• As of 10 October 2019, a total of 16 patients have been enrolled<br>• Anticipated to complete enrolment of 80 participants by December 31, 2020
Authorship eligibility guidelines and any intended use of professional writers	• We confirmed that the authorship followed the uniform requirements for manuscripts submitted to biomedical journals.<br>• Intended use of professional writers: Nil

Supplementary File 2

RESEARCH ADVISORY COUNCIL

Serious UNEXPECTED Adverse Event (SUAE) Report Form

Please complete this form for each unexpected, serious, adverse event and submit to the RAC within two working days of the occurrence.

RAC #____________________________ MRN: ________________

PI: ______________________________

Date of serious unexpected adverse event: ____

Description of the event including outcome, and timing in relation to procedure, therapy, etc:

____

____

____

____

Relationship of the event to the subject's participation in the research project:

Definitely related ☐
May be related ☐
Not related ☐

If the SUAE is related to participation in this study, please check one of the following:

☐ The possibility of this SUAE is listed in the consent form and therefore the consent form does not need to be modified.

☐ The consent form has been modified and two copies are enclosed – one with all revisions highlighted, and one clean copy to be stamped with REC approval.

☐ The SUAE was possibly related to the study, however the consent form does not need to be modified because:

____

____

____

Signature and I.D. # of P.I.: ____

Extension: ___________ MBC: _____

Date of Report: ____________________________

<u>A serious adverse event</u>" (or adverse experience) or reaction is any untoward medical occurrence that results in death, is life-threatening or potentially life-threatening, requires inpatient hospitalization or prolongation of hospitalization, results in permanent or significant disability/incapacity, results in a congenital anomaly/birth defect, or the investigator considers significant.
"<u>Unexpected</u>" is any adverse event that is not listed in the consent form and/or the proposal and includes events that are unexpected in its occurrence, severity and/or frequency.

To be completed by ORA
SUAE no. for this project:________
SUAE no. for this subject ________

Supplementary File 2

TO BE COMPLETED BY CHAIRMAN OF ☐REC ☐CRC

RECOMMENDATION:

☐ NO ACTION NEEDED UNTILL DISCUSSED BY THE FULL COMMITTEE AT THE NEXT SCHEDULED MEETING

☐ SUSPEND ENROLLMENT UNTIL DISCUSSED BY THE FULL COMMITTEE

☐ MORE INFORMATION REQUIRED (please specify)

____

____

☐ OTHER RECOMMENDATION:

____

____

Signature of Chairman: ______________________

Date: ___________________________________
